# Notes and News

**Published:** 1899-05-06

**Authors:** 


### Notes and News.

(Collected and Communicated.)

### HOSPITAL MEETINGS.

French Hospital.—Festival Dinner on Saturday, May 6th, at the Hotel Cecil.

North London Hospital for Consumption.—Festival Dinner at seven p.m., on Wednesday, May 10th, at the Hotel Cecil, Sir H. Harben presiding.

London School of Tropical Medicine (Seamen's Hospital, Greenwich) —Festival Dinner at seven p.m., on Wednesday, May 10th, at the Hotel Cecil, Mr. Chamberlain presiding.

Hospital for Diseases of the Skin, Stamford Street.—Annual Meeting at five p.m., on Wednesday, May 10th.

Belgrave Hospital for Children.—Special Meeting at the Mansion House, at three p.m., on Friday, May 12th, in furtherance of the proposal to reconstitute and transfer to another site the above hospital.

Paddington Green Children's Hospital.—Annual Meeting at five p.m., on Thursday, June 1st.

Mr. R. Brudenell Carter, F.R.C.S., has been appointed by the Council of the Society of Arts as their Trustee of the Chadwick Trust, in the room of the late Sir Douglas Galton, K.C.B.

Richmond, in Yorkshire, has just become possessed of a cottage hospital. The institution, which is the district Jubilee Memorial, has been opened by the Marchioness of Zetland. The building has been erected on land leased at a nominal rental from Lord Zetland, who provided also a cottage rent free for the residence of a nurse, the cottage being joined by a corridor with the main building.

As a token of sympathy and appreciation his friends have presented Dr. J. R. Thomson with a cheque for £1,081, to permanently associate his memory with the Royal Victoria Hospital at Bournemouth, where he has worked so long and so zealously. Dr. Thomson had been laid up for several months through a bicycle accident, an incident chosen by his friends to demonstrate the esteem in which they hold him. Dr. Thomson has placed the gift in the hands of the treasurer of the hospital, to be added to the endowment fund.

The advantage of union among medical men has been well illustrated lately in America. An attempt was made to amend the medical practice law in Nebraska so as to permit the practice of osteopathy by men without medical education or knowledge. The Bill amending the existing law found many supporters in the State Senate, but was in the end defeated. This good result was brought about mainly through the exertions of a league which had been established for the defence of the interests of the medical practitioners in the State.

A cottage hospital to serve as a memorial to the late Marquis of Drogheda has been erected at Curragh in Ireland. The site has been given by the Crown. £1,000 was collected principally amongst those connected with the turf, and subscriptions from the same source of £200 have been promised. The hospital is one of Humphrey's iron buildings. This form of building is most suitable for temporary erections, but it is unusual to construct such an institution as a memorial hospital in this manner.

Hull has for some time been suffering from a prevalence of small-pox, and the alarm felt has been heightened by the arrival of the steamship "Port Darwin," from Alexandria, with five patients suffering from the disease on board. The patients were the children of an English minister, who himself had succumbed to the disease on the voyage. The whole family of the minister and the steward who had waited upon them were removed to the hospital on the arrival of the ship, which was immediately fumigated.

An interesting fact has recently been brought into public notice. This is that the Birmingham General Hospital is part proprietor of the Theatre Royal in that city. The hospital entered into partnership in 1821, securing a large number of shares in the theatre company for the purpose of exercising some control, as a certain number of festival performances were held in the theatre for the benefit of the institution. Quite recently the hospital has received as much as £600 per annum from the dividends. Now the theatre is to be converted into a limited liability company, and under the new arrangement the hospital will own 1,800 shares of £10 each, bearing interest at 4 per cent. on the present basis.

The continued decrease in the birth-rate in France is regarded as very serious by those interested in the welfare of their country. The matter rests in the hands of individuals, and individuals have not yet learnt that it is a first duty to look to the welfare of the majority before their own. It would seem that the Government, which cannot fail to be alive to the facts, should assist rather than place obstacles in the way of those who might maintain larger families than they do. A tax on all bachelors, decreasing in proportion on the birth of each child when they marry has been suggested as a more effectual method of increasing the population of France than the present procedure, which serves to increase the taxes on those who maintain large households.

The annual report of the Porthcawl Convalescent Home exposes a weakness in respect to the admission of patients which is common to all institutions following the ticket system. At the institution in question it has been found that patients will approach more than one subscriber at once, and obtain several admission tickets, thus enabling them to extend their stay beyond the period provided for through the possession of one ticket. Such a proceeding should not be possible. Subscribers should safeguard the use of their tickets by sending them when asked for direct to the authorities for the use of the patient indicated; and, although it may be necessary in some cases to limit the duration of the period for which each ticket provides, it should be left to the medical man attending the institution to extend the period in cases where such an action is necessary, and thus those and only those who needed it would receive the benefit of a long sojourn.

